# Acute Effects of Proprioceptive Neuromuscular Facilitation on Peak Torque and Muscle Imbalance

**DOI:** 10.3390/jfmk3040063

**Published:** 2018-12-06

**Authors:** Cassio V. Ruas, Ryan T. McManus, Claudio M. Bentes, Pablo B. Costa

**Affiliations:** 1 Centre for Exercise and Sports Science Research (CESSR), School of Medical and Health Sciences, Edith Cowan University, Joondalup, WA 6027, Australia; 2 Exercise Physiology Laboratory, Department of Kinesiology, California State University, Fullerton, CA 92831, USA; 3 Clinical Research in Women’s Health, Fernandes Figueira Hospital, Oswaldo Cruz Foundation, Rio de Janeiro 22250-020, Brazil

**Keywords:** stretching, strength, eccentric, injury risk

## Abstract

Background: The effects of proprioceptive neuromuscular facilitation (PNF) stretching on muscle imbalance are not fully understood. The aim of this study was to examine the acute effects of PNF stretching on knee extension and flexion peak torque (PT), as well as the conventional and functional hamstrings to quadriceps (H:Q) ratios. Methods: Fifteen men (age = 22 ± 1 years; body mass = 76 ± 12 kg; height = 176 ± 7 cm) and fifteen women (age = 22 ± 2 years; body mass = 63 ± 8 kg; height = 161 ± 5 cm) performed concentric quadriceps and hamstrings, and eccentric hamstrings muscle actions at different angular velocities (60, 180, and 300°·s^−1^ concentric; 60 and 180°·s^−1^ eccentric) before and after a bout of PNF stretching, and a control condition. Results: Neither PNF or control conditions affected concentric PT or H:Q ratios (*p* > 0.05), apart from knee extension at 60°·s^−1^ in men (*p* = 0.001). However, there was a reduction in hamstrings eccentric PT in both control and PNF conditions for men and women (*p* = 0.003). Conclusions: PNF stretching of the hamstrings may not adversely affect the H:Q ratios, and consequently not negatively affect injury risk associated with muscular strength imbalances.

## 1. Introduction

Stretching has been typically recommended as part of athletic and recreational pre-performance activities and warm-up [1,2,3]. Several studies have demonstrated the importance of stretching on increasing flexibility and improving performance [4,5,6]. Among the most common types of stretching used for this purpose are the dynamic stretching (DS), static stretching (SS), and proprioceptive neuromuscular facilitation (PNF) [6,7,8,9]. Recent evidence has shown both DS and SS may also cause adverse effects on performance and might even increase injury risk if performed immediately prior to exercise and sport events [10,11,12,13]. This occurs because stretching may lead to alterations in neural factors and/or the mechanical components of the skeletal muscle contraction, compromising strength and affecting muscle imbalance [11,12]. For instance, static stretching may change the length-tension relationship, muscle stiffness, and viscoelastic properties of the musculotendinous unit, which can lead to stretching-induced strength decrements [14]. Additionally, dynamic stretching has been suggested to lead to changes in neural factors such as greater electromechanical delay resulting from the increased slack in the musculotendinous unit, reducing force direct transmittal from the muscle to the bone [11]. However, the potential adverse effects of PNF performed immediately before exercise on muscle imbalance are not known.

The hamstrings-to-quadriceps (H:Q) ratio has been used in many studies to describe muscle strength functionality, detect muscle imbalance, and to screen for lower extremity injury prevention [15,16,17,18,19]. This ratio can indicate potential risk of lower-extremity ligament and muscle injuries due to failure of the hamstrings to produce enough counter force to decelerate high anterior tibial shear or rotation in extended knee actions [15,17,18]. The H:Q ratio can be classified as either conventional or functional ratio [12,15,17,18,19]. The conventional ratio is determined by the balance between hamstrings and quadriceps maximal concentric strength, where values below 0.6 (60%) are indicative of increased lower-extremity injury risk [15,18,19]. The functional ratio has been described to more closely approximate muscle functionality, as it considers the deceleration of the leg by comparing hamstrings eccentric to quadriceps concentric strength, where values below 1.0 (100%) suggest high lower-extremity injury risk [15,17,18,19]. However, the long-standing assumption of these values have been previously questioned, as H:Q ratio may be altered according to the sports specific tasks and skills performed by different athletic population [13,18,20]. For instance, Magalhães et al. [21] found that H:Q ratios were different between volleyball and soccer players, as well as between sexes and those with different training levels. Additionally, Ruas et al. [18] found that a large sample of professional soccer players had similar functional ratios across different field positions, which were below the literature normative value of 1.0 (100%), indicating increased risk for future injury in the season. However, the authors concluded that specific normative values should be created for different sports to avoid overestimation of injury risk, since the values found in their study may actually represent the necessary functional balance for the performance and demands of the game. Therefore, decreases in conventional and functional H:Q ratio, even if still within the considered “normal range”, may be a concern of increased muscle imbalance, which can indicate increased risk for hamstrings strain and/or ACL tears [18,19].

The DS consists of controlled movements through active joint range of motion (ROM), including callisthenic and running drill actions (i.e., change-of direction, forward and lateral movements) [22]. The SS involves moving a limb to its maximal ROM or point of discomfort, and then holding the stretched position for a selected period of time (e.g., 15–60 s) [14,23]. PNF involves agonist muscles performing a voluntary contraction followed by a static stretch, leading to gains in ROM by reducing the reflexive components that causes muscle contraction [2,24,25]. Costa et al. [11,12] demonstrated both SS and DS decreased hamstrings and quadriceps strength, resulting in a decrease in conventional and functional H:Q ratios. Based on these results the authors recommended caution on using SS and DS immediately prior to athletic activities due to their potential adverse effects on muscle performance and injury risk markers [2,24]. However, little is known regarding the PNF effects on strength and muscle imbalance.

Many athletes and fitness enthusiasts seek increases in ROM using PNF stretching technique during pre-performance activities but wish to avoid possible related decreases in performance and associated muscle imbalances. In addition, no studies have investigated the effects of PNF on the H:Q ratios. Therefore, the aim of this study was to examine the acute effects of PNF stretching on knee extension and flexion peak torque (PT) as well as the conventional and functional H:Q ratios. Our hypothesis was that since PNF differs from SS and DS as it involves both short duration static stretching and submaximal voluntary activation, there will be less stretch-induced decreases in quadriceps and hamstrings force transmittal, which will not adversely affect the H:Q ratios.

## 2. Materials and Methods

### 2.1. Participants

Fifteen men (mean ± SD; age = 22.0 ± 1.2 years; body mass = 76.2 ± 11.7 kg; height = 176.5 ± 7.4 cm) and fifteen women (age = 22.1 ± 1.9 years; body mass = 62.6 ± 8.4 kg; height = 160.9 ± 5.5 cm) volunteered for this study. This sample size was determined using G*Power 3.1 (Institute for Experimental Psychology, Dusseldorf, Germany) based on an alpha level of 0.05 and power of 0.80, resulting in 30 participants in total. Participants were recruited by e-mail, advertisements, and by invitation of individuals of the university and community. For this, they were required to be physically active (performing physical activity for at least 30 min three days per week) and free of injuries of the knee, hip, and ankle joints for at least six months prior to participation in the study. Prior to the start of testing, all subjects read and signed an informed consent form based on the Declaration of Helsinki of ethical principles for medical research involving human participants. This study was approved by the University Ethics Committee for the Protection of Human Subjects.

### 2.2. Study Design

A repeated measures design (pre- vs. post-PNF) was used to investigate the acute effects of PNF stretching on knee extensor and flexor concentric PT, knee flexor eccentric PT, and the conventional and functional H:Q ratios. Participants visited the laboratory 3 times separated by at least 48 h. The first visit consisted of a familiarization and orientation session, while the following visits were the randomized experimental trials. In the familiarization session, participants were measured for height on a wall-mounted stadiometer and mass on a digital scale. Following this, they were familiarized with the PNF stretching protocol consisting of four assisted 6-s isometric hamstrings muscle actions and voluntary unilateral concentric knee extension and flexion strength test performed on an isokinetic dynamometer (as described in the following sections). During the experimental trials, participants completed either the stretching or control condition. For the control condition, participants sat quietly for six minutes. The PNF stretching and isokinetic tests were performed on the dominant leg, which was determined based on the participant’s kicking preference. 

### 2.3. Stretching Intervention

For the PNF stretching protocol, participants were asked to lie supine on a mat with the non-dominant thigh flexed at the hip and dominant knee fully extended. Participants were then asked to perform four assisted 6-s isometric hamstrings muscle actions at approximately 60% of perceived maximal effort followed by a static stretch of the hamstring’s muscles for 30-s hold durations. For this, whilst securing the contralateral thigh and leg, the investigator passively flexed the dominant thigh of the participants by pushing against their posterior leg and heel toward their head. The isometric contraction intensity of 60% for PNF was based on previous research showing that this intensity is just as effective as 100%, making the stretching exercise more comfortable and decreasing any risk of contraction-induced injury risk [26,27]. This was defined by the subject’s own perception of 60% of their maximal voluntary isometric contraction. A 30-s rest period was provided between repetitions. The number of repetitions was based on a previous study investigating the effect of short PNF stretching on strength decrements [28].

### 2.4. Isokinetic Peak Torque Assessment

Maximal voluntary unilateral concentric knee extension and flexion, as well as maximal eccentric knee flexion muscle actions were measured on an isokinetic dynamometer (Humac Norm CSMi, Stoughton, MA, USA). Participants were seated on the machine and positioned with velcro straps tightly placed across their thighs and chest to avoid superfluous movement of lower and upper body. The axis of rotation of the dynamometer was aligned with the lateral condyle of the dominant knee, and the lower leg was attached to the lever arm. A warm-up was performed before testing consisting of three submaximal repetitions at increasing intensities of approximately 25, 50, and 75% of the participants’ perceived maximum effort at each velocity. Following this, participants performed three maximal concentric knee extension and flexion, as well as maximal eccentric knee flexion muscle actions at randomly ordered velocities (60, 180, and 300°·s^−1^ concentric; 60 and 180°·s^−1^ eccentric) through 90° of total ROM, from 90° of knee flexion to 0° of knee extension. For the concentric test, participants were asked to “push” and “pull” the lever arm as hard and fast as possible throughout the entire ROM. For the eccentric test, participants were asked to resist the movement of the machine pulling back the lever arm as hard and fast as possible throughout the entire ROM. Isokinetic testing was performed before and after a bout of PNF stretching, or a control condition. The time between PNF and torque measurements was around 5 to 10 min. Subjects were tested 48 h to 168 h between testing sessions, and all testing sessions were conducted approximately on the same time of day within ± 2 h. High reliability has previously been found in the literature for hamstring and quadriceps concentric (ICC > 0.92) [29] and eccentric peak torque (ICC > 0.80) [30] tests. Conventional H:Q ratios were calculated by dividing the highest hamstrings concentric PT by the highest quadriceps concentric PT. Functional H:Q ratios were calculated by dividing the highest hamstrings eccentric PT by the highest quadriceps concentric PT.

### 2.5. Statistical Analyses

Five separate four-way mixed-factorial (time [pre- vs. post-stretching] × condition [stretching vs. control] × velocity [60 vs. 180 vs. 300°·s^−1^] × sex [men vs. women]) ANOVAs were performed to analyze the PT (i.e., concentric quadriceps PT, concentric hamstrings PT and eccentric hamstrings PT) and H:Q ratio data (i.e., conventional ratio and functional ratio). When appropriate, lower order two-way and one-way repeated measured ANOVA, paired sample *t*-tests, and Bonferroni corrected pairwise comparisons were conducted for follow up analyses. IBM SPSS Statistics version 22.0.0.0 (SPSS Inc., Chicago, IL, USA) was used for all statistical analyses. An alpha level of *p* ≤ 0.05 was considered statistically significant for all comparisons.

## 3. Results

**Figure 1:**
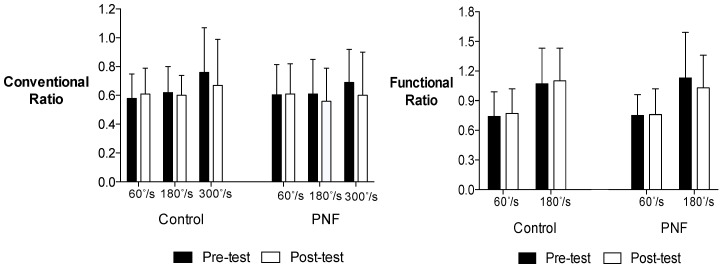
Mean values ± SD of the conventional and functional H:Q ratios for men.

**Figure 2:**
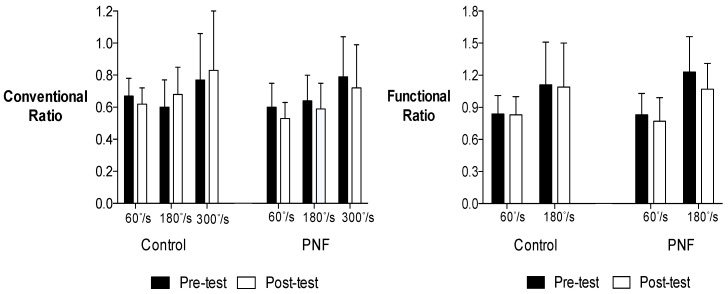
Mean values ± SD of the conventional and functional H:Q ratios for women.

Table 1 and Table 2 present the means and standard deviations (SD) for quadriceps and hamstrings concentric PT, and hamstrings eccentric PT for men and women. Figure 1 and Figure 2 present the means and SD for the conventional and functional H:Q ratios data for men and women.

### 3.1. Quadriceps Peak Torque

For quadriceps concentric PT, there was no four-way interaction for time × condition × velocity × sex (*p* = 0.302) and no three-way interaction for time × condition × velocity (*p* = 0.271), condition × velocity × sex (*p* = 0.720), or time × condition × sex (*p* = 0.805). In addition, there was no two-way interaction for condition × velocity (*p* = 0.493), time × condition (*p* = 0.218), condition × sex (*p* = 0.921), or time × sex (*p* = 0.374). However, there was an interaction for time × velocity × sex (*p* = 0.016). Two-way ANOVAs decomposed by sex indicated an interaction for time × velocity for men (*p* = 0.020) and a significant main effect for velocity (*p* < 0.001) for women. Follow-up *t*-test comparisons revealed PT at 60°/s decreased from pre- to post-test for men (*p* = 0.001). Post-hoc comparisons revealed PT decreased as angular velocity increased for women (*p* < 0.001). 

### 3.2. Hamstrings Peak Torque

For hamstrings concentric PT, there was no four-way interaction for time × condition × velocity × sex (*p* = 0.116), and no three-way interaction for time × condition × velocity (*p* = 0.400), time × velocity × sex (0.146), or time × condition × sex (*p* = 0.414). In addition, there was no two-way interaction for condition × velocity (*p* = 0.736), time × velocity (*p* = 0.238), time × condition (*p* = 0.180), condition × sex (*p* = 0.984), or time × sex (*p* = 0.156). However, there was an interaction for condition × velocity × sex (*p* = 0.05). Two-way ANOVAs decomposed by sex indicated there was an interaction for condition × velocity for women (*p* = 0.047), and a main effect for velocity for men (*p* < 0.01). Follow-up post-hoc comparisons revealed PT decreased as velocity increased in both conditions for men and women (*p* < 0.001). 

For hamstrings eccentric PT, there was no four-way interaction for time × condition × velocity × sex (*p* = 0.628), and no three-way interaction for time × condition × velocity (*p* = 0.650), condition × velocity × sex (*p* = 0.793), time × velocity × sex (*p* = 0.508), or time × condition × sex (*p* = 0.647). In addition, there were no two-way interaction for condition × velocity (*p* = 0.348), time × velocity (*p* = 0.830), time × condition (*p* = 0.818), velocity × sex (*p* = 0.391), condition × sex (*p* = 0.907), or time × sex (*p* = 0.458). However, there were significant main effects for velocity (*p* = 0.014) and time (*p* = 0.003). Follow-up post-hoc comparisons revealed PT decreased as angular velocity increased (*p* = 0.014), and that PT decreased from pre- to post-test (*p* = 0.003) for both men and women.

### 3.3. Hamstrings-to-Quadriceps Ratio

For the conventional ratio, there was no four-way interaction for time × condition × velocity × sex (*p* = 0.341), and no three-way interaction for time × condition × velocity (*p* = 0.370), condition × velocity × sex (*p* = 0.184), or time × condition × sex (*p* = 0.144). In addition, there were no two-way interaction for condition × velocity (*p* = 0.470), time × velocity (*p* = 0.345), velocity × sex (*p* = 0.307), condition × sex (*p* = 0.634), or time × sex (*p* = 0.515). However, there was an interaction for time × velocity × sex (*p* = 0.017). Two-way ANOVAs decomposed by sex indicated there was a significant main effect for velocity for women (*p* = 0.04). Follow-up post-hoc comparisons revealed conventional ratio increased as angular velocity increased for women (*p* = 0.014). For men, the percent changes from pre-test to post-test comparisons were 5.17% (60°·s^−1^), −3.23% (180°·s^−1^) and −11.84% (300°·s^−1^) for the control condition, and 0.83% (60°·s^−1^), −8.20% (180°·s^−1^) and −13.04% (300°·s^−1^) for the PNF stretching condition. For women, the percent changes from pre-test to post-test comparisons were −7.46% (60°·s^−1^), 13.33% (180°·s^−1^), 7.79% (300°·s^−1^) for the control condition, and −11.67% (60°·s^−1^), −7.81% (180°·s^−1^), −8.86% (300°·s^−1^) for the PNF stretching condition.

For the functional ratio, there was no four-way interaction for time × condition × velocity × sex (*p* = 0.833), and no three-way interaction for time × condition × velocity (*p* = 0.204), condition × velocity × sex (*p* = 0.475), time × velocity × sex (*p* = 0.983), or time × condition × sex (*p* = 0.871). In addition, there were no two-way interaction for condition × velocity (*p* = 0.600), time × velocity (*p* = 0.105), time × condition (*p* = 0.062), velocity × sex (*p* = 0.762), condition × sex (*p* = 0.850), or time × sex (*p* = 0.157). However, there was a main effect for velocity (*p* < 0.001). Follow-up post-hoc comparisons revealed functional ratio increased as angular velocity increased for both men and women (*p* < 0.001). For men, the percent changes from pre-test to post-test comparisons were 4.05% (60°·s^−1^) and 2.80% (180°·s^−1^) for the control condition, and 1.33% (60°·s^−1^) and −8.85% (180°·s^−1^) for the PNF stretching condition. For women, the percent changes from pre-test to post-test comparisons were −1.19% (60°·s^−1^) and −1.80% (180°·s^−1^) for the control condition, and −7.23% (60°·s^−1^) and −13.01% (180°·s^−1^) for the PNF stretching condition.

## 4. Discussion

The aim of this study was to examine the effects of PNF stretching on knee extension and flexion peak torque (PT) and the conventional and functional H:Q ratios. The main results indicated neither the current stretching protocol or a control condition affected concentric PT, with the exception of knee extension at the 60°·s^−1^ in men. However, there was a reduction in hamstrings eccentric PT in both control and PNF conditions for men and women. Additionally, neither conditions had an adverse effect on the H:Q ratios. These findings suggest PNF stretching of the hamstrings may not adversely affect the H:Q ratios, and consequently not negatively affect injury risk associated with muscular strength imbalances.

A plethora of studies have reported DS and SS can negatively affect hamstrings and/or quadriceps PT when performed immediately prior to exercise [11,12,23,31,32]. However, previous research describing the effects of PNF on strength performance has shown conflicting results [28,33,34,35,36,37]. While a few studies have reported PNF can be detrimental to vertical jump, and isometric as well as dynamic strength performance [33,34,35], others have found PNF does not induce decreases in strength, muscle activation, or functional performance [28,36,37], and is equally or more effective in improving flexibility compared to other types of stretching [36]. Part of these discrepancies might be because stretching-induced strength decrements may depend on the intensity, volume, training status of participants, and time-elapsed between stretching and post-test [10,32]. Additionally, PNF elicits different results if administered with stretches of short duration or several static stretching performed for longer than 30 s, as short duration stretches may not be sufficient to affect neural drive or force decrements [28,37]. For instance, Place et al. [28] reported PNF performed with short contraction and stretching phases (i.e., 5 s each) did not lead to decrements in jumping performance. Furthermore, quadriceps maximal strength decreased similarly after PNF and a control condition involving 2-min walking, but knee flexion and hip extension active ROM did not change after either condition. Likewise, Young and Elliot [38] demonstrated that while 3 repetitions of 15 s of static stretching affected drop jump performance, PNF with 3 repetitions of 5 s of isometric contraction followed by 15 s of stretch on quadriceps, gluteus, and plantar flexors did not negatively affect vertical jump scores. These findings are partially in accordance with our results as we found a lack of condition × time interactions for hamstrings and quadriceps PT, which indicates there was no difference between PNF (involving four repetitions of 6-s isometric contractions followed by static stretch holds of 30 s) and control conditions. Therefore, PNF involving sets of 6-s isometric contractions followed by static stretching of no longer than 30-s static stretch holds may be an alternative warm-up strategy to avoid stretch-induced decreases in performance.

Improved balance between hamstrings and quadriceps, measured by H:Q ratio, is essential for performance as an imbalance may be represent a risk factor for sustaining injuries such as hamstrings strain and ACL tears [15,17,18]. However, both DS and SS have previously been shown to decrease the H:Q ratio, subsequently increasing risk of lower-extremity injuries during exercise [11,12]. Costa et al. [12] reported conventional and functional ratios were differently affected by hamstrings and quadriceps SS. While the conventional ratio was reduced after hamstrings-only SS, the functional ratio decreased after both hamstrings and quadriceps SS as well as quadriceps-only SS. Similarly, in a second study [11], the authors found hamstrings and quadriceps DS decreased concentric and eccentric hamstrings PT, which led to decreases in both conventional and functional ratios. Stretching did not cause adverse effect on quadriceps strength, leading to reduced H:Q ratios, which may be related to the different force producing capabilities, cross sectional area, ROM capacity, and muscle architecture between hamstrings and quadriceps muscle groups [11,13]. Costa et al. [11] stated that stretching may cause an increase in the electromechanical delay of the hamstrings due to the greater slack in the musculotendinous unit, which impacts how force is transmitted directly from the contractile component to the bone. 

Contrary to these results, PNF stretching did not adversely affect the H:Q ratios, which is related to the non-significant stretch-induced decreases in hamstrings force transmittal. This indicates PNF might pose a better alternative to be included as a mean of injury prevention during athletic and recreational pre-performance warm-up activities compared to SS and DS. Place et al. [28] found that M-wave characteristics (which may indicate muscle excitability) and peak twitch (which may determine the final stages of excitation-contraction coupling) of the knee extensor muscles were preserved after PNF performed with short durations. This may explain why hamstrings strength was not affected after our PNF intervention, and not affecting the H:Q ratios. However, the aforementioned study did not report differences in quadriceps ROM, which questions whether PNF stretching of greater duration is needed to induce changes on stretch perception, viscoelastic properties of the musculotendinous unit, or proprioceptive information, leading to alterations on muscle force transmittal. Additionally, Minshull et al. [36] reported PNF flexibility training had superior preservation of electromechanical delay of the hamstrings compared to passive stretching (i.e., application of external force to stretch the muscle). Perhaps since PNF involves static maximal and submaximal voluntary activation [39], contractile-induced adaptations may preclude flexibility-induced changes due to the electromechanical delay [36], favoring dynamic joint stability and reducing musculoskeletal injury risk.

## 5. Conclusions

The results indicated there was no difference between PNF stretching or a control condition on hamstrings and quadriceps peak torque or the H:Q ratios. These findings suggest PNF stretching of the hamstrings may not adversely affect the H:Q ratios, and consequently might not negatively affect injury risks associated with muscular strength imbalances.

## Figures and Tables

**Table 1:** Mean values ± SD of quadriceps and hamstrings concentric PT, and hamstrings eccentric PT for men.

Men	Pre-Test			Post-Test			Percent Change		
**Control Condition**	**60°·s^−1^**	**180°·s^−1^**	**300°·s^−1^**	**60°·s^−1^**	**180°·s^−1^**	**300°·s^−1^**	**60°·s^−1^**	**180°·s^−1^**	**300°·s^−1^**
Quadriceps Concentric PT (N·m)	207.67 ± 34.76	139.20 ± 32.46	99.73 ± 22.72	192.73 ± 37.53 *	130.73 ± 31.52	102.53 ± 29.65	−7.19	−6.08	2.81
Hamstrings Concentric PT (N·m)	120.07 ± 36.02	84.13 ± 23.23	73.13 ± 23.90	115.33 ± 30.29	77.67 ± 20.09	66.20 ± 28.40	−3.95	−7.68	−9.48
Hamstrings Eccentric PT (N·m)	150.93 ± 50.10	145.00 ± 43.47	-	145.00 ± 42.15 *	139.87 ± 40.10 *	-	−3.93	−3.54	-
**PNF Stretching Condition**	**60°·s^−1^**	**180°·s^−1^**	**300°·s^−1^**	**60°·s^−1^**	**180°·s^−1^**	**300°·s^−1^**	**60°·s^−1^**	**180°·s^−1^**	**300°·s^−1^**
Quadriceps Concentric PT (N·m)	210.07 ± 54.27	132.80 ± 33.55	106.40 ± 23.37	196.53 ± 49.92 *	139.53 ± 38.43	103.13 ± 26.69	−6.45	5.07	−3.07
Hamstrings Concentric PT (N·m)	121.40 ± 30.78	76.87 ± 22.91	70.67 ± 22.04	115.33 ± 30.35	73.80 ± 19.90	59.06 ± 27.17	−5.00	−3.99	−16.43
Hamstrings Eccentric PT (N·m)	152.13 ± 38.76	139.40 ± 38.84	-	144.80 ± 46.19 *	137.13 ± 37.45 *	-	−4.82	−1.63	-

* Denotes significant decrease from pre- to post-condition.

**Table 2:** Mean values ± SD of quadriceps and hamstrings concentric PT, and hamstrings eccentric PT for women.

Women	Pre-Test			Post-Test			Percent Change		
**Control Condition**	**60°·s^−1^**	**180°·s^−1^**	**300°·s^−1^**	**60°·s^−1^**	**180°·s^−1^**	**300°·s^−1^**	**60°·s^−1^**	**180°·s^−1^**	**300°·s^−1^**
Quadriceps Concentric PT (N·m)	114.33 ± 21.16	79.20 ± 16.38	58.07 ± 16.32	108.33 ± 21.23	74.00 ± 15.69	53.00 ± 12.90	−5.25	−6.57	−8.73
Hamstrings Concentric PT (N·m)	76.67 ± 21.61	46.87 ± 13.71	43.33 ± 17.15	67.40 ± 18.23	50.07 ± 14.00	43.40 ± 20.50	−12.09	6.83	0.16
Eccentric Hamstring PT (N·m)	95.87 ± 27.11	91.79 ± 21.24	-	90.13 ± 27.69 *	83.86 ± 25.10 *	-	−5.99	−8.64	-
**PNF Stretching Condition**	**60°·s^−1^**	**180°·s^−1^**	**300°·s^−1^**	**60°·s^−1^**	**180°·s^−1^**	**300°·s^−1^**	**60°·s^−1^**	**180°·s^−1^**	**300°·s^−1^**
Quadriceps Concentric PT (N·m)	116.07 ± 30.87	76.27 ± 20.67	57.20 ± 19.17	115.13 ± 23.61	76.73 ± 17.96	57.13 ± 13.87	−0.81	0.60	−0.12
Hamstrings Concentric PT (N·m)	69.13 ± 20.90	48.40 ± 14.73	44.80 ± 17.70	60.93 ± 16.97	44.00 ± 11.61	40.93 ± 17.83	−11.86	−9.09	−8.64
Hamstrings Eccentric PT (N·m)	94.27 ± 24.52	90.0 ± 19.23	-	85.40 ± 16.16 *	79.73 ± 14.81 *	-	−9.41	−11.41	-

* Denotes significant decrease from pre- to post- condition.
